# Supplementary material for: A method for allocating low-coverage sequencing resources by targeting haplotypes rather than individuals
Source: Genet Sel Evol. 2017 Oct 25;49:78. doi: 10.1186/s12711-017-0353-y (PMC5655873; doi:10.1186/s12711-017-0353-y)
Supplement: Supplementary file 1 — Additional file 1: Figure S1. Expected haplotype imputation accuracy against the accumulated haplotype sequencing coverage, as estimated using a novel population-based imputation method (Battagin and Hickey, unpublished). A description of the prototype algorithm that was developed for the imputation of consensus haplotypes under the LCSeq approach and the simulated results on which the AlphaSeqOpt method is based. We generated 1x sequence data for the sires from a simulated population. The x-axis represents the expected accumulated coverage that each haplotype would receive. The y-axis represents the percentage of alleles phased and imputed for each haplotype. The imputation accuracy increased with the accumulated haplotype coverage until it plateaued. Haplotypes with a sequencing coverage of 10x accumulated from 20 individuals sequenced at 1x were imputed to the whole population with an accuracy of 0.88. Haplotypes with a sequencing coverage of 15x or 20x accumulated from 30 or 40 individuals sequenced at 1x were imputed to the whole population with an accuracy of 0.93 or 0.97, respectively. For accurate inference of a consensus haplotype, a certain amount of sequencing coverage must be accumulated. According to the results above, 10x or 15x could be good target coverages for the haplotypes [file 12711_2017_353_MOESM1_ESM.pdf]

Additional file 1

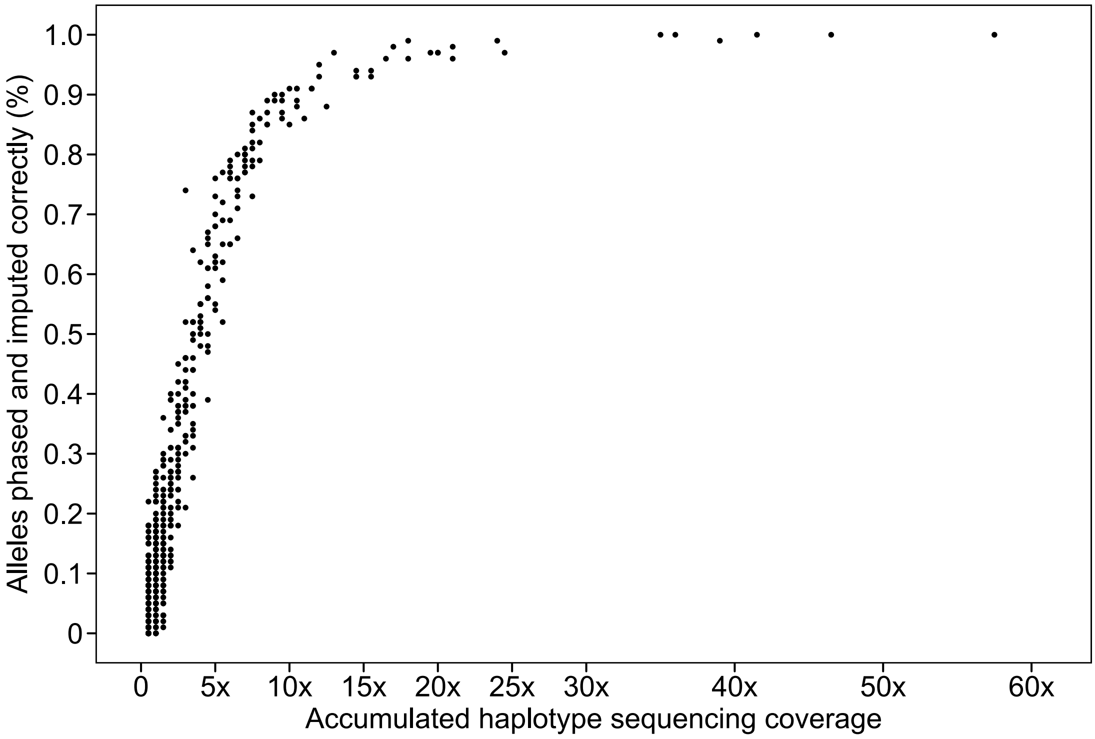

Figure S1. Expected haplotype imputation accuracy against the accumulated haplotype sequencing coverage, as estimated using a novel population-based imputation method (Battagin and Hickey, unpublished).

A prototype algorithm was developed for the imputation of consensus haplotypes under the LCSeq approach (Battagin and Hickey, unpublished). The prototype algorithm follows the following four steps: (1) Phasing of the alleles at homozygous loci. Each individual that shares a particular haplotype is expected to be homozygous at different loci, which allows the phasing of large proportions of the shared haplotypes. (2) The phased alleles of the haplotypes that several individuals share are put together to build a consensus haplotype. In case of conflict the most common allele can be used or the allele can be left unimputed depending on the quality of the read information collected. The consensus haplotype is propagated to all individuals that share that particular haplotype so that alleles at heterozygous loci can also be phased. (3) Phasing of the alleles of the complementary haplotypes. (4) Iteration through steps 2 and 3 until convergence.

We simulated a population of 46,889 individuals with marker array genotypes using AlphaSim [21,22] as described in the Materials and Methods section. The marker array genotypes were used to define the haplotypes as described in the Materials and Methods section. We then generated 1x sequence data for the 1,819 sires of that population. The sequence data was simulated by sampling sequencing reads for each individual and loci using a Poisson-gamma distribution to model sequenceability of each loci and number of reads for each individual at each loci, as described in: G. Gorjanc, J.F. Dumasy, S. Gonen, C. Gaynor, R. Antolin, & J.M. Hickey. 2017. Potential of low-coverage genotyping-by-sequencing and imputation for cost-effective genomic selection in biparental segregating populations. *Crop Science*, 57: 1404-1420.

Figure S1 shows the results for the 914 haplotypes in a core. The x-axis represents the expected accumulated coverage that each haplotype would receive (effectively, half the number of times the haplotype was observed in the sires sequenced at 1x). The accumulated haplotype coverage was 2.0x in average and it ranged from 0x, for haplotypes not carried by the sires, to 57.5x, for the most common haplotype. The y-axis represents the percentage of alleles phased and imputed correctly for each haplotype. The imputation accuracy increased with the accumulated haplotype coverage until it plateaued. Haplotypes with a sequencing coverage of 10x accumulated from 20 individuals sequenced at 1x were imputed to the whole population with an accuracy of 0.88. Haplotypes with a sequencing coverage of 15x or 20x accumulated from 30 or 40 individuals sequenced at 1x were imputed to the whole population with an accuracy of 0.93 or 0.97, respectively. For accurate inference of a consensus haplotype, a certain amount of sequencing coverage must be accumulated. According to the results above, 10x or 15x could be good target coverages for the haplotypes.
